# Contact tracing following outbreak of Ebola virus disease in urban settings in Nigeria

**DOI:** 10.11604/pamj.supp.2017.27.1.12565

**Published:** 2017-05-28

**Authors:** Olufunmilayo Ibitola Fawole, Mahmood Muazu Dalhat, Meeyoung Park, Casey Daniel Hall, Patrick Mboya Nguku, Peter Adebayo Adewuyi

**Affiliations:** 1 Nigeria Field Epidemiology and Training Program, Abuja, Nigeria; 2 Department of Epidemiology and Medical Statistics, College of Medicine, University of Ibadan, Ibadan, Nigeria; 3 Rollins School of Public Health, Emory University, Atlanta, USA

**Keywords:** Public health, epidemiology, Ebola virus disease, contact tracing

## Abstract

An outbreak of Ebola virus disease occurred in Nigeria between July and September 2014. Contact tracing commenced in Lagos, and extended to Port Harcourt and Enugu as the outbreak continued to spread. A total of 899 contacts were traced. Contact tracing enhanced immediate identification of symptomatic contacts, some of whom eventually became cases. Contact tracing could be challenging in urban cities. However, use of electronic technology, adequate logistics, and highly skilled personnel enhanced the tracing of contacts to facilitate the successful containment of the outbreak. Nigeria was certified to be Ebola free on 21st October 2014. Ebola virus surveillance needs to be maintained to ensure the disease has been contained and to prevent future outbreaks. This case study aims to help trainees to review concepts, apply skills, and address challenges for contact tracing based on the experience of the Nigerian Field Epidemiology Training Network during the 2014 Ebola virus disease outbreak.

## How to use this case study

**General instructions:** this case study in applied epidemiology allows students to practice applying epidemiologic skills in the classroom setting to address real-world public health problems. The case study is used as a vital component of an applied epidemiology curriculum, rather than as a stand-alone tool. It is suited to reinforcing principles and skills already covered in a lecture or in background reading. Ideally, 1 to 2 instructors facilitate the case study for 10 to 15 students in a classroom or conference room setting. The instructor should direct participants to read aloud a paragraph or two, going around the room and giving each participant a chance to read. When a participant reads a question, the instructor directs participants to engage in discussions or exercises as recommended in the note for the instructor in the instructor’s guide. Questions are answered by participants serially based on how they are seated to ensure active participation. Sometimes, the instructor’s guide may recommend splitting the class into groups to play different roles or assume different sides of the discussion when answering the question. All questions involve group discussion and reflection of the answer. As a result, participants learn from each other, not just from the instructors.

**Audience:** the primary audience includes residents in Field Epidemiology Training Programs (FETPs) and Field Epidemiology and Laboratory Training Programs (FELTPs). The secondary audience includes health professionals (such as disease surveillance and notification officers and other field officers) working in the African region in government and non-governmental health organisations who are interested in this topic.

**Prerequisites:** before using this case study, case study participants should have received lectures or other instructions on contact tracing or read the guidelines on contact tracing.

**Materials needed:** a white board or flip chart is recommended for recording responses. A projector will also be required to project to the class a flow diagram.

**Level of training and associated public health activity:** basic – contact tracing, i.e. this case study is for participants who may not or may already have an understanding of how to conduct an outbreak investigation such as tier 1 and 2 of the CDC Applied Epidemiology Competencies (http://www.cdc.gov/AppliedEpiCompetencies/).

**Time required:** approximately 2-3 hours

**Language:** English

## Case study material

- Download the case study student guide (PDF - 2.42 MB)
- Request the case study facilitator guide
